# The use of multi-criteria method in the process of threat assessment to the environment

**DOI:** 10.1038/s41598-021-97939-4

**Published:** 2021-09-14

**Authors:** Elwira Zajusz-Zubek, Zygmunt Korban

**Affiliations:** 1 Department of Air Protection, Faculty of Energy and Environmental Engineering, Silesian University of Technology, 22B Konarskiego St., 44-100 Gliwice, Poland; 2 Department of Safety Engineering, Faculty of Mining, Safety Engineering and Industrial Automation, Silesian University of Technology, 2 Akademicka St., 44-100 Gliwice, Poland

**Keywords:** Ecology, Environmental sciences, Engineering

## Abstract

Measurements of the content of trace elements, including toxic and carcinogenic metals, in various fractions of particulate matter PM are an important element of environmental monitoring and research involving their impact on human health. The article presents the measurement results of atmospheric composition of suspended dust (PM_10_), respirable fraction (PM_2.5_) and submicron particulate matter (PM_1_) collected with the Dekati PM_10_ cascade impactor. Samples were collected in the vicinity of four working power plants (from 28 May to 23 September 2014) and four coking plants (from 4 May to 28 August 2015) in Upper Silesia, Poland. The qualitative and quantitative analysis of the solutions: arsenic (As), cadmium (Cd), cobalt (Co), chromium (Cr), mercury (Hg), manganese (Mn), nickel (Ni), lead (Pb), antimony (Sb) and selenium (Se) obtained for individual fractions was performed by inductively coupled plasma mass spectrometry, using the apparatus ICP-MS. The research results were used to determine a synthetic assessment of the threat to the anthropogenic environment and for the preparation of the ranking of the measured points.

## Introduction

Based on statistical data, in the last 20–30 years the emissions to the atmosphere from main anthropogenic sources have been significantly reduced both in Europe and in Poland. One of the most dangerous pollutants in atmospheric air in many cities is the particulate matter (PM)^1^. Epidemiological studies demonstrate that negative health effects in humans apply not only to people exposed to raised mass concentrations of particulate matter, but they also result from its chemical, physical and biological properties. The assessment of occupational risk (including health risk) is the subject of analyzes and research conducted by a wide range of institutions (among others: Comité Européen de Normalisation (CEN); The International Agency for Research on Cancer (IARC); National Institute for Occupational Safety and Health (NIOSH)) and people^2–5^. Toxicological studies have shown some cellular mechanisms of PM adverse effects, for instance, reactive oxygen species (ROS) formation leads to oxidative potential (OP), DNA damage, mutagenicity, and stimulation of inflammatory cytokine production. PM exposure induces oxidative stress and inflammation in the airways. The increase in the level of ecological and health risk is also associated with the presence of heavy metals. It is caused mainly by anthropogenic processes, often reinforced natural events (conditions)^6–9^. In recent years, worldwide and in Poland, there has been a growing interest in the impact of trace elements (including toxic and carcinogenic metals) contained in various fractions of particulate matter (PM) on environmental pollution and human health (in terms of the aerodynamic diameter of dust particles, we can distinguish: total suspended particles TSP, inhalable dust PM_10_ (particles with an aerodynamic diameter ≤ 10 μm), respirable fraction PM_2.5_ (dust with an aerodynamic diameter ≤ 2.5 μm) and submicron particulate matter PM_1_ (particles with an aerodynamic diameter ≤ 1 μm)^10^. The negative health effects increase principally due to the exposure to respirable aerosol fractions, which may cause long-term and cumulative effects in the form of chronic bronchitis, cardiovascular diseases, lung cancer and even death^2,3^. The International Agency for Research on Cancer (IARC) has assigned suspended dust to group I, i.e. substances with proven carcinogenic activity, and has classified the most dangerous trace elements into the following groups:

- group 1—elements which are carcinogenic for humans,
- group 2—elements possibly carcinogenic for humans,
- group 3—elements non-classified as carcinogenic for humans.

With respect to elements selected for the research (As, Cd, Co, Cr, Hg, Mn, Ni, Pb, Sb and Se), according to the above-mentioned classification, group 1 comprises: arsenic, chromium, cadmium and nickel; group 2: cobalt and lead; and group 3 contains selenium. According to the Agency for Toxic Substances and Disease Registry, mercury and manganese are classified as toxic elements^11,12^, but the group of toxic elements includes also antimony. Moreover, antimony is probably carcinogenic^13^. At the same time, in numerous research programs, e.g. EMEP (The European Monitoring and Evaluation Program), UNEP (United Nations Environment Program), mercury together with lead and cadmium are classified as priority pollutants.

In Poland, the concentration of PM_10_ and PM_2.5_ in the air of urban-industrial agglomerations reaches much higher levels than in Western European countries. High concentrations of particulate matter in the air are currently one of the most important public health problems. Particulate matter particles comprise heavy metals, which are contained in dust grains, or are present on their surface^14^.

The concentration and composition of particulate matter in a specific place depend on many natural and anthropogenic factors, such as its local or regional sources, meteorological conditions or geographic location^15–17^.

In contrast to the commonly performed emission measurements carried out at the outlet of the emitter, as part of the present study research was conducted in the vicinity of industrial pollution emission sources.

The sampling sites of air (measurement points located in the surroundings of the power plants (P_1_ $\div$ P_4_ and the coking plants K_1_ $\div$ K_4_) were interpreted as points in the multidimensional space. The research results were used to determine a synthetic assessment of the threat to the anthropogenic environment, thanks to which we can focus preventive actions not only on information resulting from taking into account individual describing parameters independently, but also all monitored harmful (onerous) factors present in the environment. In the computational layer, one of the solution methods of multi attribute decision making, the so-called development measure method was applied.

In Poland, the significance of the problem is greater due to the long-term perspective of using coal for energy purposes. This problem affects all countries where energy policy is based on coal, such as China, India and North America. The global coking industry has a slightly upward trend, while in Poland it remains rather stable. Hence, the research undertaken in this paper is of importance beyond national issues.

## Scope and methodology of research

The research was carried out on the basis of direct measurements in the surroundings of four selected working coal-fired power plants and four working coking plants. The samples of suspended dust PM_10_, respirable fraction PM_2.5_ and submicron particulate matter PM_1_ were collected in the surroundings of power generation facilities and in the surroundings of coking plants.

### Location of measurement points

**Figure 1:**
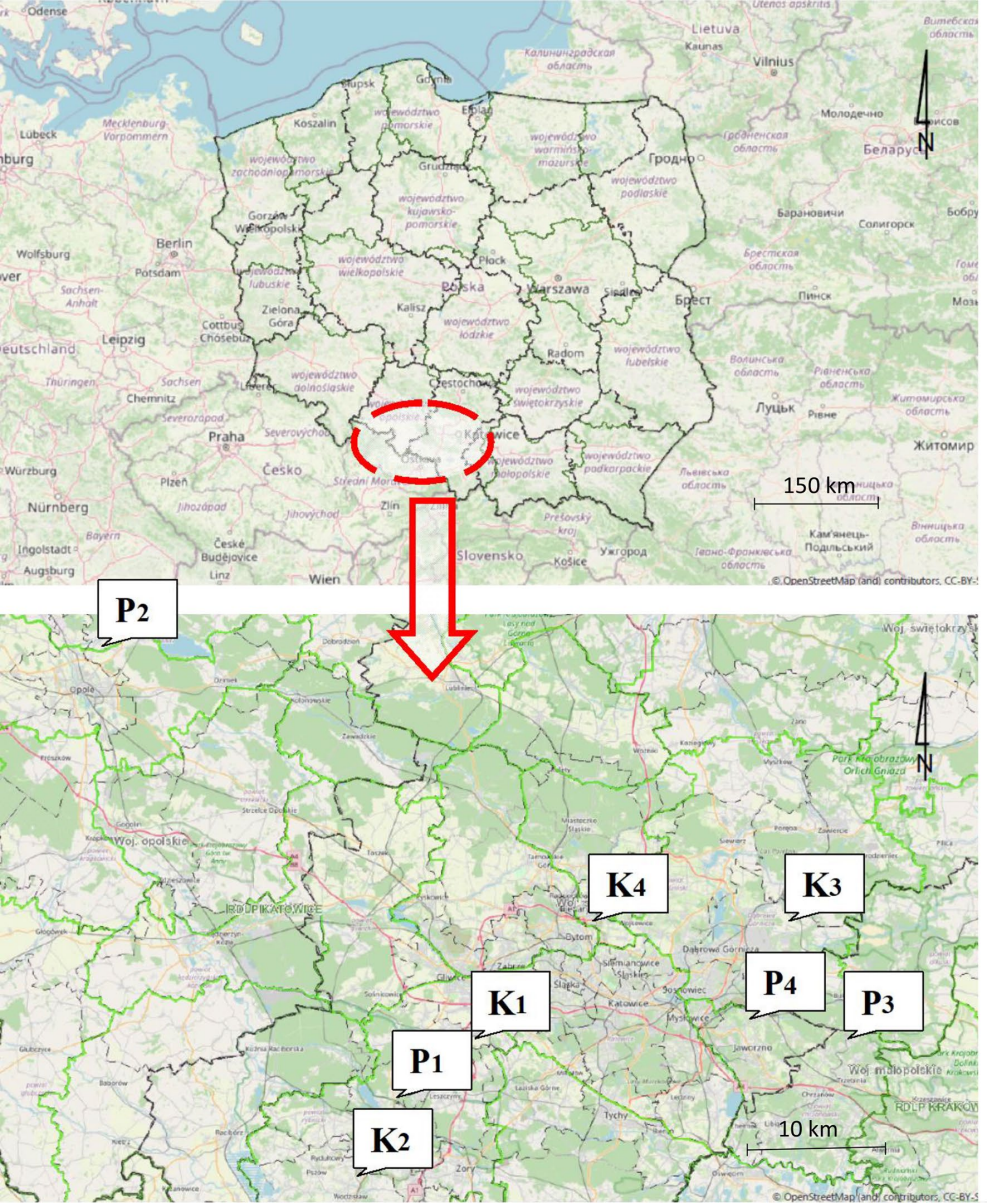
Location of the sampling sites (the map was generated based on data from the BDL^18^ website).

The location of the measurement points was selected in southern Poland, around the selected four working coal-fired power plants and four working coking plants. The sampling points in the surroundings of the power plant (P_1_, P_2_, P_3_ and P_4_) and the coking plant (K_1_, K_2_, K_3_ and K_4_) were located at the distance of approximately 2 km to the north-east from the respective object (Fig. 1).

The location of the measurement points was a compromise, taking into account the representativeness of the receptor, the possibility to connect the testing equipment and the consent of the property owners. To eliminate the impact of a heating season, and especially that of low emissions, presented in the studies by^19^, the measurement sessions were carried out only in the summer season. The samples of particulate matter were collected on a weekly basis, with 4 sessions at one site. The methodology applied in this work is presented in^20,21^. The location of measurement sites:

- point P_1_: 50° 08′ 37.87″ N; 18° 32′ 15.76″ (Golejów—a suburban district of Rybnik in the Śląskie Voivodeship, in the vicinity of a working power plant with a capacity of 1775 MW; population:
- 2 300);
- point P_2_: 50° 45′ 35.41″ N; 17° 56′ 20.43″ E (Świerkle—a rural area in the Opolskie Voivodeship (Dobrzeń Wielki commune) near a working power plant with a capacity of 1,492 MW; population: 520);
- point P_3_: 50° 12′ 33.46″ N; 19° 28′ 28.77″ E (Czyżówka—rural area in the Małopolskie Voivodeship (commune of Trzebinia) near a working power plant with a capacity of 786 MW; population: 700);
- point P_4_: 50° 13′ 48.90″ N; 19° 13′ 24.45″ E (suburbs of Jaworzno (Śląskie Voivodeship) in the vicinity of a 1,345 MW power plant; number of inhabitants: 95 500);
- K_1_ point: 50° 10′ 11.36″ N; 18° 40′ 34.35″ E (Czerwionka—Leszczyny in the Śląskie Voivodeship, in the vicinity of a small coking plant; number of inhabitants: 27 300);
- K_2_ point: 50° 3′ 19.76″ N; 18° 30′ 21.69″ E (Popielów—a suburban district of Rybnik in the Śląskie Voivodeship, surrounded by a small working coking plant; population:3 300);
- K_3_ point: 50° 21′ 24.08″ N; 19° 21′ 37.46″ E (Łęka—Dąbrowa Górnicza district, in the Śląskie Voivodeship, surrounded by a large coking plant; number of inhabitants: 700);
- K_4_ point: 50° 21′ 0.47″ N; 18° 53′ 15.44″ E (Bytom—a city in the Śląskie Voivodeship, a small coking plant located on the outskirts of the city; population: 174 700).

The state of air pollution with particulate matter in the area investigated in the study is affected by various local sources of pollution emissions. At the measurement sites P_1_, P_2_, P_3_ and P_4_, the emissions are mainly from power plant chimneys, but also from auxiliary processes, i.e. coal storage and its transport. In addition, the recorded emissions are also influenced by other industrial plants operating in the vicinity of the measurement sites, domestic and municipal sector and the impact of automotive industry. The measurement sites K_1_, K_2_, K_3_ and K_4_ involve primarily the emissions accompanying the processes of coal coking as well as auxiliary processes, i.e. coal deposition, its transmission, management of products and post-production wastes. Additionally, they are affected by the emissions from industrial plants and low emission sources operating in this area, as well as the emission from the combustion of solid fuels for domestic or municipal purposes, as well as by the automotive industry.

### Sampling process

The samples of suspended dust (PM_10_), respirable fraction (PM_2.5_) and submicron particulate matter (PM1) were collected using the Dekati PM_10_ cascade impactor serial No. 6648 by Dekati (Finland) with the air flow rate of $1.8 {\mathrm{m}}^{3}/\mathrm{h}$. The impactor Dekati PM_10_ guarantees the collection of dust samples for three cutpoint diameters: 10 μm, 2.5 μm and 1 μm. For the sampling at the first, second and third stages of the impactor, polycarbonate filters were used (Nuclepore 800 203, with the diameter of 25 mm, by Whatman International Ltd., Maidstone, UK). At the fourth stage, the dust was collected on a Teflon filter for particles ≤ 1 μm in diameter (Pall Teflo R2PJ047, 47 mm in diameter, by Pall International Ltd., New York, NY, USA). The average volume of air passing through the filters was approximately 300 m^3^. The impactor’s capture efficiency was characterized by the uncertainty below 2.8%. The mass of dust collected at the individual stages of the impactor was determined by the gravimetric method, and it was referenced to the volume of passed air $\left(\mathrm{\mu g}/{\mathrm{m}}^{3}\right)$ according to the PN-EN12341^22^. All impactor samples were analysed by inductively coupled plasma mass spectrometry (ICP-MS).

The samples were collected at a height of 1.5 m from the ground, i.e. in the breathing zone for people. The respective dust fractions were collected in 7-day cycles from 28 May to 24 September 2014 (16 weeks) in the surroundings of four working coal-fired power plants and from 4 May to 28 August 2015 (16 weeks) in the surroundings of four working coking plants. The measurement campaign comprised four measurement sessions separately for each sampling site. One session comprised dust sampling at each stage of the Dekati PM_10_ cascade impactor and filters used for reference. The filters were taken back after study period and labeled during the collection process in the field and stored in the plastic containers for safe transportation and storage in laboratory for further analysis.

In each measurement session, blind filters were stored at the sampling site, but they were not subjected to exposure. The sample data were corrected from these blanks. The length of the measurement cycles was conditioned by the need to collect an appropriate amount of research material (with the aerodynamic diameter of the dust grains < 1; 1 $\div$ 2.5; 2.5 $\div$ 10 and > 10 μm). Analogous (7-day) periods of dust sampling were used in the studies by^4,23^.

Polycarbonate and Teflon filters were conditioned before and after dust collection at a temperature of 20 ± 1 °C (relative humidity 50%$\pm$ 5%) for 48 h, and then weighed on a microbalance with an accuracy of 1 $\mathrm{\mu g}$ (MXA5/1, by RADWAG, Poland).

Taking into account the measurement sessions at four sites in the surroundings of the power plant (P_1_ $\div$ P_4_) and at four sites in the surroundings of the coking plant (K_1_ $\div$ K_4_), the aggregate number of samples exceeded 450.

### Chemical analysis

The qualitative and quantitative analysis of the obtained solutions was performed by inductively coupled plasma mass spectrometry using an ICP-MS instrument (NexION 300D, PerkinElmer, Inc., Waltham, MA, USA). For all elements determined simultaneously, the same parameters of the instrument were used, which are presented in the publications^20,21,24^.

As standards for the determination of ^75^As, ^111^Cd, ^59^Co, ^53^Cr, ^200^Hg, ^55^Mn, ^60^Ni, ^206^Pb, ^121^Sb and ^82^Se, we applied the 1000 $\mathrm{\mu g}/{\mathrm{cm}}^{3}$ CertPUR ICP multi-element standard solution VI for ICP-MS by Merck, Germany. Ten repetitions were performed for all samples. The determined limits of detection (LOD) were based on 10 independent measurements for blank test. For the results obtained in that way, the mean value and the value of the standard deviation SD were calculated. The values of LOD for individual elements were determined on the basis of the dependence (1):

1 $$\mathrm{LOD}= {\mathrm{x}}_{\mathrm{sr}}+ 3\mathrm{SD}$$

where: x_śr_—mean concentration value of the element, $\mathrm{g}/{\mathrm{dm}}^{3}$, SD—standard deviation.The determination correctness of the content of the elements was verified with the use of certified reference materials: European Reference Material ERM-CZ120 and Standard Reference Material SRM 1648a (National Institute of Standards and Technology, USA). The recovery with the use of the said certified reference materials was respectively as follows: As (111% for ERM-CZ120 and 96% for SRM 1648a), Cd (97% and 105%), Co (108% and 97%), Cr (103% and 94%), Mn (106% and 100%), Ni (107% and 102%), Pb (107% and 105%) and Sb (99% and 91%). The certified reference materials did not contain Hg or Se.

## Development measure method—theoretical fundamentals

In the process of broadly understood assessment, synthetic measures determined by the application of solution methods of multi attribute decision making (MADM) are becoming increasingly important. These methods allow to build scalars (synthetic assessment indicators), taking into account the numerical values of the criteria and the differentiation of weights assigned to them, and then the generated measures enable the replacement of the entire set of features describing the object (partial evaluations) with one variable being an aggregate value^25–29^. Table 1 presents a list of selected groups of methods used in solving multi-attribute decision-making problems (MADM).

**Table 1:** Overview of selected groups of methods for solving multi-attribute decision-making problems^30,31^.

No	Group of methods	Characteristics of groups of methods
1	Methods making use of reference points	Objects (variants) are compared with abstract reference solutions: ideal (development measure method), ideal and anti-ideal (Technique for Order Preference by Similarity to Ideal Solution (TOPSIS), VIsekrzterijumskaOptimizacija and KompromisnoResenje (VIKOR)). For each object (variant), its distance from the reference solution(s) is determined
2	Additive methods	The matrix of normalized assessments is determined and the object (variant) is selected for which the sum of assessments is the highest (Simple AdditiveWeighting Method (SAW), Fuzzy Simple AdditiveWeighting Method (F-SAW)—modification of the SAW method using fuzzy numbers)
3	Analytical hierarchy methods and related methods	Independent criteria and objects (variants) are compared in pairs, which enables to create a scale vector and to order the objects (variants). Example of the method: Analytical Hierarchy Process (AHP), Analytic Network Process (ANP) which is an extension of the AHP method, Ratio Estimation in MagnitudesordeciBells to RateAlternativeswhichare Non-DominaTed (REMBRANDT), which makes use of a logarithmic scale when comparing the criteria
4	Verbal methods	Methods based mainly on qualitative parameters for which an objective aggregation model cannot be developed (ZAPROS, ZAPROS III)
5	Methods of ELECTRE family	Objects (variants) are assessed in terms of maximized criteria (each criterion is assigned a positive weight, the compliance index is determined and the condition of non-compliance lack is checked). The final result is made up by the outranking relation and the structure of the dependency graph between the objects (ELECTRE I, ELECTRE IV (it is possible to differentiate veto thresholds for different criteria), ELECTRE III (for each pair of variants, compliance index and credibility index are determined))
6	PROMETHEE methods	Objects are compared in pairs due to the adopted evaluation criteria. Preference functions are determined and the indifference and strict preference thresholds are defined. Preference flows are determined for each pair of objects (variants). Example of the method: PROMETHEE I, PROMETHEE II, PROMETHEE II + weto, EXtension of the PROMetheemethod (EXPROM)—modification of the PROMETHEE II (it includes the ideal and anti-ideal variants)
7	Interactive methods	It is assumed that the decision-maker has information to assess a single variant or a set of variants. The procedure is divided into successive iterations, and each of them consists of a computational phase and a phase of dialogue with the decision-maker. Examples of the methods: STEp Method for DiscreteDecisionMakingProblemsunderRisk (STEM-DPR), INteractiveStochasticDECisionMakingProcedure (INSDECM)

The development measure method used in the article is based on the notion of ordering binary relation and is one of the oldest methods of linear ordering. In this method, in order to determine the evaluation criteria (goodness criteria), the following are defined:

- an abstract point P_o_ which illustrates a model solution having the coordinates $\left\{{\mathrm{x}}_{\mathrm{o}1}, {\mathrm{x}}_{\mathrm{o}2}, \dots {\mathrm{x}}_{\mathrm{om}}\right\}$ meeting the conditions^32^:
  2 $$ {\text{x}}_{{{\text{oj}}}} = {\text{max x}}_{{{\text{ij}}}} ,{\text{when j}} \in {\text{S}} $$
  3 $$ {\text{x}}_{{{\text{oj}}}} = {\text{min x}}_{{{\text{ij}}}} ,{\text{when j}} \in {\text{D}} $$
  where: S—a set of stimulants (features, variables, whereof high values are desirable from the point of view of the diagnosed problem, and the low ones—undesirable); D—a set of destimulants (features, variables whereof low values, unlike in the case of stimulants, are desirable from the point of view of the diagnosed problem).
  The set of features may also be made of: nominants, i.e. variables having the character of stimulants and destimulants, and neutral features—independent variables (or variables having too weak dependence on the explained variable);
- points P_i_, which are a graphical interpretation of the objects subjected to assessment and then the distances between the individual points Pi and the point Po are determined according to the relationship:
  4 $${\mathrm{C}}_{\mathrm{io}}= \sqrt{\sum_{\mathrm{j}=1}^{\mathrm{m}}{\mathrm{\alpha }}_{\mathrm{j}}{\left({\mathrm{x}}_{\mathrm{ij}}^{,}- {\mathrm{x}}_{\mathrm{oj}}^{,}\right)}^{2}}$$
  where: ${\mathrm{x}}_{\mathrm{ij}}^{,}$—standardized coordinates of the point P_i_; ${\mathrm{\alpha }}_{\mathrm{j}}$—significance (rank) of the j-th partial feature determined on the basis of an expert opinion survey, or in line with the coefficient of variation.

The basic condition enabling the determination of the above-mentioned measure is to standardize the output variables, which has the following aim^33^:

- bringing the variables with different titers to comparability (postulate of additivity),
- unifying the nature of variable features (postulate of uniform preference),
- elimination of non-positive values (postulate of positivity),
- replacement of different ranges of variability of the features with constant ranges (postulate of stability of the range or postulate of constancy of extreme values).

Bringing the variables with different titers to comparability (implementation of the additivity postulate) can be based on^33–36^:

- ranking of variables,
- quotient transformations,
- standardization of variables,
- unitization of variables.

In the article, in the process of variables normalization, we use quotient transformations where, depending on the nature of the variables, the reference point of the features (variables) is the maximum value, or the minimum value in the set of features:

5 $${\mathrm{x}}_{\mathrm{ij}}^{\mathrm{^{\prime}}}=\frac{{\mathrm{x}}_{\mathrm{j}}^{\mathrm{min}}}{{\mathrm{x}}_{\mathrm{ij}}}, {\mathrm{X}}_{\mathrm{j}}\in \mathrm{D}, {\mathrm{x}}_{\mathrm{ij}}\ne 0$$

6 $${\mathrm{x}}_{\mathrm{ij}}^{\mathrm{^{\prime}}}=\frac{ {\mathrm{x}}_{\mathrm{ij}}}{{\mathrm{x}}_{\mathrm{j}}^{\mathrm{max}}}, {\mathrm{X}}_{\mathrm{j}}\in \mathrm{S}, {\mathrm{x}}_{\mathrm{j}}^{\mathrm{max}}\ne 0$$

The final value of the development measure mi was calculated from the relationship:

7 $${\mathrm{m}}_{\mathrm{i}}=1- {\mathrm{C}}_{\mathrm{io}}/{\mathrm{C}}_{\mathrm{io max}}$$

where $${\mathrm{m}}_{\mathrm{i}} \in \le 0;1\ge $$.

An object is assumed to be more developed the closer its measure is to 1.

## Discussion of results

The sampling sites of air (measurement points) located in the surroundings of the power plants and the coking plants were interpreted as points in the multidimensional space, whereof coordinates (dimensions) correspond respectively to the average concentrations of the contents of:

- arsenic, ng/m^3^ (j = 1),
- cadmium, ng/m^3^ (j = 2),
- cobalt, ng/m^3^ (j = 3),
- chromium, ng/m^3^ (j = 4),
- mercury, ng/m^3^ (j = 5),
- manganese, ng/m^3^ (j = 6),
- nickel, ng/m^3^ (j = 7),
- lead, ng/m^3^ (j = 8),
- antimony, ng/m^3^ (j = 9),
- selenium, ng/m^3^ (j = 10),
- dust PM_10_, µg/m^3^ (j = 11),
- dust PM_2.5_, µg/m^3^ (j = 12),
- dust PM_1_, µg/m^3^ (j = 13)

which were recorded from 28 May to 23 September 2014 and from 4 May to 28 August 2015^24^. The measurement results are summarized in the form of a matrix:

8 $$\left[\begin{array}{ccc}{\mathrm{x}}_{11}& \cdots & {\mathrm{x}}_{1\mathrm{j}}\\ \vdots & \ddots & \vdots \\ {\mathrm{x}}_{\mathrm{i}1}& \cdots & {\mathrm{x}}_{\mathrm{ij}}\end{array}\right]$$

where: i—object under assessment; j—coordinate of the object.

The summary of input data (mean concentration values) are presented in the matrix notation:

$$\left[\begin{array}{c}0.98\\ 0.73\\ \begin{array}{c}0.76\\ 1.09\\ \begin{array}{c}0.89\\ 0.87\\ \begin{array}{c}1.20\\ 1.57\end{array}\end{array}\end{array}\end{array} \begin{array}{c} 1.08\\ 1.21\\ \begin{array}{c} 0.67\\ 1.00\\ \begin{array}{c} 1.03\\ 0.97\\ \begin{array}{c} 1.84\\ 3.88\end{array}\end{array}\end{array}\end{array}\begin{array}{c} 0.47\\ 0.47\\ \begin{array}{c}0.07\\ 0.08\\ \begin{array}{c}0.12\\ 0.23\\ \begin{array}{c}0.37\\ 0.54\end{array}\end{array}\end{array}\end{array} \begin{array}{c} 64.14\\ 64.23\\ \begin{array}{c}25.10\\ 25.11\\ \begin{array}{c}28.23\\ 28.57\\ \begin{array}{c}80.27\\ 81.26\end{array}\end{array}\end{array}\end{array} \begin{array}{c} 1.58 \\ 1.56\\ \begin{array}{c}0.12\\ 0.07\\ \begin{array}{c}0.74\\ 0.76\\ \begin{array}{c}1.69\\ 1.71\end{array}\end{array}\end{array}\end{array} \begin{array}{c}13.22\\ 13.92\\ \begin{array}{c} 8.19\\ 11.58\\ \begin{array}{c}10.82\\ 22.95\\ \begin{array}{c}26.13\\ 29.30\end{array}\end{array}\end{array}\end{array}\begin{array}{c} 6.91\\ 5.82\\ \begin{array}{c} 3.07\\ 0.67\\ \begin{array}{c} 2.63\\ 1.96\\ \begin{array}{c} 16.40\\ 14.72\end{array}\end{array}\end{array}\end{array}\begin{array}{c} 13.79\\ 21.84\\ \begin{array}{c} 23.88\\ 25.35\\ \begin{array}{c} 22.95\\ 19.67\\ \begin{array}{c} 53.35\\ 183.04\end{array}\end{array}\end{array}\end{array}\begin{array}{c} 1.54\\ 1.04\\ \begin{array}{c} 0.58\\ 1.99\\ \begin{array}{c} 1.70\\ 1.17\\ \begin{array}{c} 1.25\\ 2.91\end{array}\end{array}\end{array}\end{array}\begin{array}{c} 20.53\\ 21.19\\ \begin{array}{c} 2.05\\ 1.78\\ \begin{array}{c} 9.24\\ 9.51\\ \begin{array}{c} 1.78\\ 1.80\end{array}\end{array}\end{array}\end{array} \begin{array}{c}18.03\\ 15.92\\ \begin{array}{c}15.78\\ 15.58\\ \begin{array}{c}23.57\\ 22.61\\ \begin{array}{c}13.30\\ 28.23\end{array}\end{array}\end{array}\end{array} \begin{array}{c} 15.52 \\ 12.56\\ \begin{array}{c}12.75\\ 11.55\\ \begin{array}{c}18.29\\ 17.75\\ \begin{array}{c} 7.85\\ 21.63\end{array}\end{array}\end{array}\end{array} \begin{array}{c}12.78\\ 8.68\\ \begin{array}{c} 8.74\\ 8.13\\ \begin{array}{c}14.09\\ 13.79\\ \begin{array}{c} 3.85\\ 17.35\end{array}\end{array}\end{array}\end{array}\right]$$

All the examined features have the character of destimulants (there is a negative correlation with the dependent variable, which is the pollution level of anthropogenic environment).

The input data after normalization are compiled in the form of a matrix:

$$\left[\begin{array}{c}0.745\\ 1.000\\ \begin{array}{c}0.961\\ 0.670\\ \begin{array}{c}0.820\\ 0.839\\ \begin{array}{c}0.608\\ 0.465\end{array}\end{array}\end{array}\end{array} \begin{array}{c} 0.620\\ 0.554\\ \begin{array}{c}1.000\\ 0.670\\ \begin{array}{c}0.650\\ 0.691\\ \begin{array}{c}0.364\\ 0.173\end{array}\end{array}\end{array}\end{array}\begin{array}{c} 0.149\\ 0.149\\ \begin{array}{c}1.000\\ 0.875\\ \begin{array}{c}0.583\\ 0.304\\ \begin{array}{c}0.189\\ 0.130\end{array}\end{array}\end{array}\end{array} \begin{array}{c}0.391\\ 0.391\\ \begin{array}{c}1.000\\ 1.000\\ \begin{array}{c}0.889\\ 0.879\\ \begin{array}{c}0.313\\ 0.309\end{array}\end{array}\end{array}\end{array} \begin{array}{c}0.044\\ 0.045\\ \begin{array}{c}0.583\\ 1.000\\ \begin{array}{c}0.095\\ 0.092\\ \begin{array}{c}0.041\\ 0.041\end{array}\end{array}\end{array}\end{array} \begin{array}{c}0.620\\ 0.588\\ \begin{array}{c}1.000\\ 0.707\\ \begin{array}{c}0.757\\ 0.357\\ \begin{array}{c}0.313\\ 0.280\end{array}\end{array}\end{array}\end{array}\begin{array}{c} 0.097\\ 0.115\\ \begin{array}{c} 0.218\\ 1.000\\ \begin{array}{c} 0.255\\ 0.342\\ \begin{array}{c} 0.041\\ 0.046\end{array}\end{array}\end{array}\end{array}\begin{array}{c} 1.000\\ 0.631\\ \begin{array}{c} 0.577\\ 0.544\\ \begin{array}{c} 0.601\\ 0.701\\ \begin{array}{c} 0.258\\ 0.075\end{array}\end{array}\end{array}\end{array}\begin{array}{c} 0.377\\ 0.558\\ \begin{array}{c} 1.000\\ 0.291\\ \begin{array}{c} 0.341\\ 0.496\\ \begin{array}{c} 0.464\\ 0.199\end{array}\end{array}\end{array}\end{array}\begin{array}{c} 0.087\\ 0.084\\ \begin{array}{c} 0.868\\ 1.000\\ \begin{array}{c} 0.193\\ 0.187\\ \begin{array}{c} 1.000\\ 0.989\end{array}\end{array}\end{array}\end{array} \begin{array}{c}0.738\\ 0.835\\ \begin{array}{c}0.843\\ 0.854\\ \begin{array}{c}0.564\\ 0.588\\ \begin{array}{c}1.000\\ 0.471\end{array}\end{array}\end{array}\end{array} \begin{array}{c} 0.506\\ 0.625\\ \begin{array}{c} 0.616\\ 0.680\\ \begin{array}{c} 0.429\\ 0.442\\ \begin{array}{c} 1.000\\ 0.363\end{array}\end{array}\end{array}\end{array} \begin{array}{c} 0.301\\ 0.444\\ \begin{array}{c} 0.441\\ 0.474\\ \begin{array}{c} 0.273\\ 0.279\\ \begin{array}{c} 1.000\\ 0.222\end{array}\end{array}\end{array}\end{array}\right]$$

The weights of criteria ${\mathrm{\alpha }}_{\mathrm{j}}$ were determined using the variation coefficient ${\mathrm{v}}_{\mathrm{s}}^{\mathrm{j}}$:

9 $${\mathrm{v}}_{\mathrm{s}}^{\mathrm{j}}=\frac{{\mathrm{s}}_{\mathrm{j}}}{{\mathrm{z}}_{\mathrm{srj}}^{\mathrm{^{\prime}}}}$$

where: ${\mathrm{z}}_{\mathrm{srj}}^{\mathrm{^{\prime}}}$—arithmetic mean for j = 1, 2, …13

10 $${\mathrm{\alpha }}_{\mathrm{j}}=\frac{{\mathrm{v}}_{\mathrm{s}}^{\mathrm{j}}}{\sum_{\mathrm{j}=1}^{13}{\mathrm{v}}_{\mathrm{s}}^{\mathrm{j}}}$$

The summary of the values of the coefficients of variation and weights of criteria is presented in Table 2.

**Table 2:** Ranking of objects and summary of development measure values.

$${\mathbf{v}}_{\mathbf{s}}^{\mathbf{j}}$$												
j = 1	j = 2	j = 3	j = 4	j = 5	j = 6	j = 7	j = 8	j = 9	j = 10	j = 11	j = 12	j = 13
0.273	0.707	0.654	0.509	0.676	0.465	0.912	1.249	0.464	0.980	0.268	0.299	0.395
$${{\varvec{\upalpha}}}_{\mathbf{j}}$$												
j = 1	j = 2	j = 3	j = 4	j = 5	j = 6	j = 7	j = 8	j = 9	j = 10	j = 11	j = 12	j = 13
0.035	0.090	0.083	0.065	0.086	0.059	0.116	0.159	0.059	0.125	0.034	0.038	0.050

The aggregated values determined on the basis of the formula (6) (the values of development measure) were used to determine the ranking of the examined objects (Table 3).

**Table 3:** Ranking of objects and summary of development measure values.

Object i	m_i_	Ranking of objects
1	0.147	6
2	0.150	5
3	0.523	2
4	0.593	1
5	0.250	3
6	0.241	4
7	0.146	7
8	0.000	8

In the case of the diagnosed objects (power plants and coking plants), the measurement results involving the concentration of elements and the fraction of particulate matter in the air can be treated in two ways:

- as final results within the single-criteria assessment involving the hazard to the anthropogenic environment,
- as partial results within the multi-criteria assessment involving the hazard to the anthropogenic environment.

In the first case, each i-th object was assessed separately as part of the j-th criterion (each object is assessed separately on the basis of the observed concentration values of subsequent elements and fractions of particulate matter), and in the second case the measurement results are used to determine the aggregated (synthetic) final assessment of the hazard state.

Basing on the results of single-criteria assessments, rankings of objects examined in the research were prepared (Table 4).

**Table 4:** Single-criterion assessment—rankings of objects examined in the research.

Ranking place	Assessment criterion (j)												
	As	Cd	Co	Cr	Hg	Mn	Ni	Pb	Sb	Se	PM_10_	PM_2.5_	PM_1_
1	P_2_	P_3_	P_3_	P_3_	P_4_	P_3_	P_4_	P_1_	P_3_	P_4_	K_3_	K_3_	K_3_
2	P_3_	K_2_	P_4_	P_4_	P_3_	K_1_	K_2_	K_2_	P_2_	K_3_	P_4_	P_4_	P_4_
3	K_2_	P_4_	K_1_	K_1_	K_1_	P_4_	K_1_	P_2_	K_2_	K_4_	P_3_	P_2_	P_2_
4	K_1_	K_1_	K_2_	K_2_	K_2_	P_1_	P_3_	K_1_	K_3_	P_3_	P_2_	P_3_	P_3_
5	P_1_	P_1_	K_3_	P_1_	P_2_	P_2_	P_2_	P_3_	P_1_	K_1_	P_1_	P_1_	P_1_
6	P_4_	P_2_	P_1_	P_2_	P_1_	K2	P_1_	P_4_	K_1_	K_2_	K_2_	K_2_	K_2_
7	K_3_	K_3_	P_2_	K_3_	K_3_	K_3_	K_4_	K_3_	P_4_	P_1_	K_1_	K_1_	K_1_
8	K_4_	K_4_	K_4_	K_4_	K_4_	K_4_	K_3_	K_4_	K_4_	P_2_	K_4_	K_4_	K_4_

Based on the determined values of the synthetic measure, we can observe that the objects P_4_ and P_3_ had the highest, similar values of the measure mi: the first place in the ranking was occupied by the object P_4_.

(m_4_ = 0.593), the second place—the object P_3_ (m_3_ = 0.523). The following objects obtained considerably worse results: K_1_ (m_5_ = 0.250), K_2_ (m_6_ = 0.241), P_2_ (m_2_ = 0.150), P_1_ (m_1_ = 0.147) and K_3_ (m_7_ = 0.146), occupying positions 3 – 7, respectively. The last place in the ranking was occupied by the object K_4_, whose development measure value was 0.000.

The object P_4_ (i = 4) is the most developed object among the assessed objects (the value of measure mi is closest to 1.0), despite the fact that in the rankings prepared on the basis of single-criterion assessments, it took the first place only three times criterion 5: mercury concentration, criterion 7: nickel concentration and criterion 10: selenium concentration (jointly with the object K_3_)), with five leading positions of the object P_3_ (i = 3) (criterion 2: cadmium concentration, criterion 3: cobalt concentration, criterion 4: chromium concentration, criterion 6: manganese concentration and criterion 9: antimony concentration). The above state can be explained both by the places of objects in other problem areas (for five criteria: No. 3 (cobalt concentration), No. 4 (chromium concentration (jointly with the object P_3_)), No. 11 (PM_10_ dust concentration), No. 12 (PM_2.5_ dust concentration) and No. 13 (PM_1_ dust concentration)). The object P_4_ took second positions in the rankings, and the worst in his case was the seventh place in the ranking, No.9: antimony concentration), as well as by the values of rankings assigned to individual criteria (for criteria 9 (antimony concentration) and criterion 6 (manganese concentration).

Although the object K_3_ occupied the first place four times in the individual rankings (criteria 10 –13), but also the weights of criteria 11, 12 and 13 were the lowest, and the average observed concentrations of mercury and nickel were the highest among the examined objects.

The lowest value of the development measure was obtained by the object K_4_ (m_8_ = 0.000)—in this case only the concentrations of nickel (j = 7) and selenium (j = 10) were not the highest in the set of the examined objects.

## Conclusions

Apart from the improvement of living standards, industrialization and technological development also have negative connotations, which is confirmed, among others, by the state of anthropogenic environment. The above conclusion also applies to the state of the atmosphere where negative health effects are caused by both carcinogenic elements (arsenic, chromium, cadmium and nickel), by possibly carcinogenic elements (cobalt and lead), elements non-classified as carcinogenic to health (selenium), and particulate matter with its chemical, physical and biological properties (in terms of the results of environmental measurements used in the article, the WHO standards and the permissible levels in force in Polish legislation were not exceeded with regard to the concentration of suspended dust PM_10_ and contained in it carcinogenic elements as well as with respect to the concentration of the respirable fraction PM_2.5_). Currently, the problem does not involve the measurements of the above-mentioned elements, or their archiving. The problem is how to use the results of monitoring, e.g. in the process of broadly understood prevention. In the opinion of the authors, ensuring fast, up-to-date and accurate information, combined with the use of numerical information, often with the simultaneous use of IT techniques (simulation methods, active control methods, forecasting methods, etc.) can significantly affect the quality of decision-making, also aimed to improve the quality of air. The article presents the possibility of determining the ranking (classification) of objects (8 objects) and their grouping in line with the adopted criteria (13 criteria), using one of the linear ordering methods (development measure method). The obtained results of the multi-criteria assessment allow us to formulate the following:

- the objects P_4_ (m_4_ = 0.593) and P_3_ (m_3_ = 0.523) are characterized by the shortest distance from the ideal point (hypothetical point P_0_), and hence, for these objects, we can talk about the most favorable (among the assessed objects) parameters describing the anthropogenic environment;
- the least favorable parameters describing the anthropogenic environment among the assessed objects are attributed to the object K_4_ (m_8_ = 0.000), which means that it is the farthest from the ideal point;
- a similar threat to the anthropogenic environment was reported for objects P_4_ and P_3_ (group 1), K_1_ and K_2_ (group 2), as well as P_2_, P_1_ and K_3_ (group 3).

The model of information processing proposed in the article is an example of a comprehensive approach to the measurement results of environmental parameters (the information contained e.g. in databases), thanks to which we can focus preventive actions not only on information resulting from taking into account individual describing parameters independently, but also all monitored harmful (onerous) factors present in the environment (the development measure method enables practically an unlimited expansion of both the set of objects subjected to assessment (*i* → ∞) and the set of describing features / assessment criteria.

(*j* → ∞)).
